# Comparison of Postoperative Cognitive Decline Using the Mini-Mental State Examination and Montreal Cognitive Assessment After Minor Elective Surgery in Elderly

**DOI:** 10.7759/cureus.18631

**Published:** 2021-10-09

**Authors:** Ismail Aytaç, Betül Güven Aytaç, Gokhan Demirelli, Duygu Kayar Çalılı, Semih Baskan, Aysun Postacı, Nermin Göğüş

**Affiliations:** 1 Anesthesiology, Ankara City Hospital, Ankara, TUR; 2 Anesthesiology and Reanimation, Bafra State Hospital, Samsun, TUR; 3 Intensive Care, Ankara City Hospital, Ankara, TUR; 4 Anesthesiology and Critical Care, Yıldırım Beyazıt University, Ankara, TUR

**Keywords:** anesthesia, spinal, geriatrics, mmse, mini mental state examination, montreal cognitive assessment, postoperative cognitive dysfunction

## Abstract

Introduction and aim

Postoperative cognitive dysfunction (POCD) is an important complication associated with increased morbidity, mortality, and reduced quality of life. Generally, studies have focused on major surgery so there is little evidence of the incidence of cognitive dysfunction in minor surgery. We aimed to compare the Mini-Mental State Examination (MMSE) and Montreal Cognitive Assessment (MoCA) in terms of detecting cognitive decline in elderly patients after elective inguinal herniorrhaphy procedure with general or spinal anesthesia.

Material and methods

This observational study was conducted from June 2014 to March 2015 at Ankara Numune Education and Research Hospital. The type of anesthesia was determined according to the anesthesiologist’s preference who is blind to the study. Patients were grouped according to anesthesia received: general or spinal anesthesia. The MMSE and MoCA were evaluated presurgery and 24 hours after the operation.

Results

The postoperative (24th hour) MMSE scores of patients (26.23±2.77) were significantly lower than the preoperative scores (27.17±1.93) in only the general anesthesia group (p =0.003). The postoperative (24th hour) MoCA scores (22.87±3.88 for general and 23.13±4.08 for spinal anesthesia) were significantly lower than the preoperative scores (24.32±3.19 for general and 24.35±2.84 for spinal anesthesia) in both the general and spinal anesthesia groups (p =0.000 and 0.019, respectively). The incidence of postoperative cognitive dysfunction was 32.9% using the MoCA and 15.2% using the MMSE (p=0,018).

Conclusion

Early POCD is an important problem after elective minor surgeries, even with spinal anesthesia, in elderly patients. The MoCA is an alternative tool that can be more sensitive than the MMSE to identify cognitive decline in elderly patients undergoing minor surgeries under both general and spinal anesthesia.

## Introduction

Similar to the world demographic structure, the population of Turkey is rapidly aging and, by 2023, it is estimated that the number of people over 65 years of age in the country will rise to 8.87 million (10.2% of the total population) [1]. Therefore, it can be predicted that anesthesiologists will have to deal more with elderly patients in the near future. Anesthesiologists encounter many problems in elderly patients, who have reduced efficacy of physiological functions. Postoperative pulmonary, cardiovascular, and renal complications are more likely to occur and adversely affect outcomes in elderly patients [2]. In addition, postoperative cognitive dysfunction (POCD) is another frequent complication associated with increased morbidity, mortality, and reduced quality of life [2,3].

Most POCD studies have focused on cardiac or non-cardiac major surgeries, and the incidence or severity varies depending on the type of surgery, cognitive assessment time, POCD definition, and neuropsychological testing tools [4-8]. Cognitive decline is usually noticed by the patient’s family and can also be detected by comparison of preoperative and postoperative neuropsychological testing. Despite the importance of POCD, evaluation of cognition is often overlooked in elderly patients undergoing elective surgical procedures [9]. There are few studies of POCD in minor surgical procedures and outpatient cases in elderly patients, although minor surgeries account for a large proportion of the surgical burden [10-12]. Certain negative features of the hospital environment, such as noise and preoperative anxiety, may impair cognitive functions in elderly patients, even in minor and outpatient surgeries [10,11].

The Mini Mental State Examination (MMSE) is a widely used cognitive assessment tool to detect POCD in multiple cognitive domains, including orientation, registration, attention and calculation, immediate recall, language, short-term memory, and constructability. The time required for the examination is 5-15 minutes, but it has high specificity and low sensitivity to detect mild cognitive impairment [13-15]. On the other hand, the Montreal Cognitive Assessment (MoCA) is a new cognitive screening tool for multiple cognitive domains, including short-term memory, visuospatial skills, executive function, attention, concentration and working memory, language, and orientation. The MoCA has high sensitivity and specificity to detect patients with mild cognitive impairment performing in the normal range on the MMSE [15-17].

In this observational study, we aimed to compare the MMSE and MoCA in terms of detecting cognitive decline in elderly patients after elective inguinal herniorrhaphy procedure with general or spinal anesthesia. We conclude that the MoCA can detect more elderly patients with cognitive decline, even in the spinal anesthesia group.

## Materials and methods

**Figure 1:**
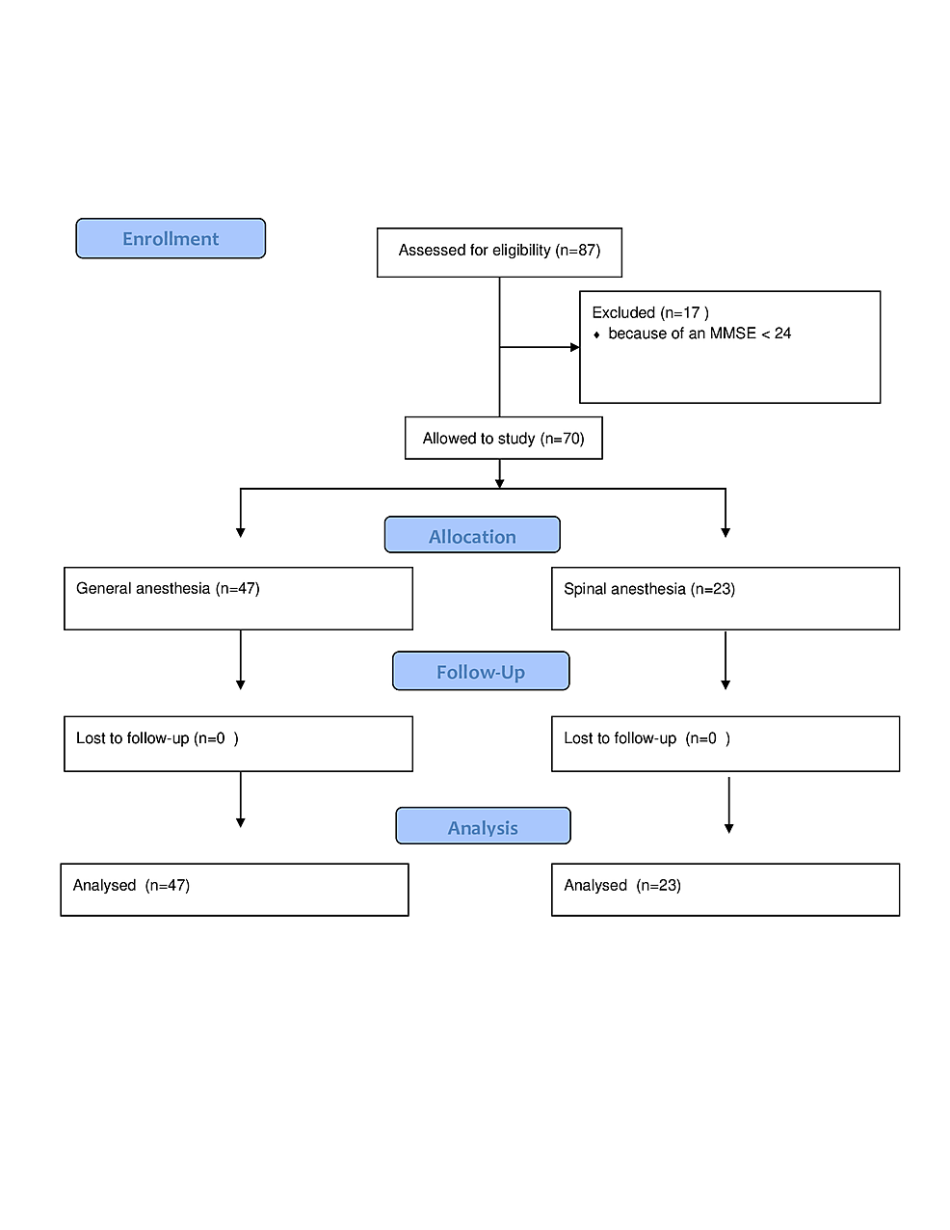
Flow chart of the study

This prospective observer-blind cohort study was conducted at Ankara Numune Education and Research Hospital between June 2014 and March 2015 after obtaining approval from the Institutional Ethics Committee (dated on 06/18/2014 and numbered E-14-216) and written informed consent from the patients (Figure 1). The inclusion criteria were fluency in Turkish, age ≥ 65, American Society of Anesthesiologists (ASA) score I-III, and being scheduled for elective inguinal herniorrhaphy. The exclusion criteria were an MMSE score < 24 in the preoperative examination, suffering from diseases of the central nervous system, use of antidepressant or antipsychotic medication, alcohol or drug abuse, and being illiterate.

The MMSE and MoCA scores were evaluated in a quiet room and recorded by a blinded researcher during the preoperative visit at a desk and at the 24th hour after the operation in quiet conditions at the sickbed. During the preoperative examination, we recorded the patient’s age, gender, ASA score, BMI, comorbidities, and information on previous anesthesia management.

The type of anesthesia was determined according to the anesthesiologist’s preference who is blind to the study. General anesthesia was induced with 1 µg/kg fentanyl and 2mg/kg propofol until loss of eyelash reflex. Maintenance was achieved by 1.5-2.5% sevoflurane in 50% N_2_O and 50% O_2_. Spinal anesthesia was conducted with 2-2.5 ml of 0.5% hyperbaric bupivacaine. Routine intraoperative monitoring data in five minutes intervals (non-invasive mean arterial blood pressure, heart rate, and pulse oximetry) were recorded from the patient’s medical file. Postoperative laboratory parameters, complications, and discharge times were also noted.

 Statistical analysis

Discrete data were expressed as numbers and percentages, while continuous variables were reported as mean and standard deviation when normally distributed, along with the median and minimum and maximum values. According to the suitability of the normal distribution of the data for the comparison of continuous variables, the t-test or Mann-Whitney U-test were used. To compare nominal data, the chi-square test or Fisher exact test were utilized. The Wilcoxon test served for the comparison of the MMSE and MoCA measurements before and after anesthesia in each anesthesia group. The analysis was performed with SPSS 11.5 (2002, SPSS Inc., Chicago, Illinois) and p < 0.05 was accepted as the statistical significance limit.

## Results

In the preoperative period, we evaluated 87 patients, among which 17 (19.5%) were excluded because of an MMSE < 24. The demographic characteristics of the patients are summarized in Table 1. The general and spinal anesthesia groups were similar in terms of mean age, BMI, distribution of gender, ASA scores, and comorbidity percentages (p > 0.05).

**Table 1:** Demographics of patients † t-test/chi-square test: Fisher’s exact test Continuous data are presented as mean ± standard deviation and categorical data as number (percentage). ASA: American Society of Anesthesiologists

	General anesthesia (n = 47)	Spinal anesthesia (n = 23)	p-value†
Age (year)	69.72 ± 4.74	69.43 ± 4.16	0.809
BMI (kg/ m^2 ^)	27.59 ± 2.98	27.0 ± 3.18	0.459
Gender, Female	8 (17.4)	5 (21.7)	0.663
Gender, Male	38 (82.6)	18 (78.3)	
ASA I	1 (2.3)	1 (4.3)	0.128
ASA II	41 (95.3)	19 (82.6)	
ASA III	1 (2.3)	3 (13)	
Hypertension	16 (34)	10 (43.5)	0.443
Diabetes mellitus	3 (6.4)	5 (18)	0.104
Heart disease	7 (14.9)	7 (30.4)	0.202
Renal disease	1 (2.1)	0 (0)	1.000

The intraoperative characteristics are summarized in Table 2. In the spinal anesthesia group, the duration of operation was significantly longer compared to the general anesthesia group (p = 0.003). The intraoperative mean arterial pressure, SpO2, heart rate levels, and postoperative length of hospital stay were similar between groups.

**Table 2:** Intraoperative characteristics of the groups † t-test/chi-square test: Fisher’s exact test or Mann-Whitney U tests were used for comparison where appropriate. * Significance level was p<0.01

	General anesthesia (n = 47)	Spinal anesthesia (n = 23)	p-value†
Duration of operation (min)	60 (30- 90)	75 (45- 120)	0.003*
Mean arterial pressure (mmHg)	97 (81- 145)	102 (83- 133)	0.234
SpO_2_	98 (95- 100)	98 (94- 100)	0.410
Heart rate	82 (60−104)	78 (68−108)	0.460
Length of hospital stay (days)	1 (0−16)	4 (0−10)	0.080

The postoperative (24th hour) MMSE scores were significantly lower than the preoperative scores only in the general anesthesia group (p = 0.003), while the postoperative (24th hour) MoCA scores were lower than the preoperative scores in both the general and spinal anesthesia groups (p = 0.000 and 0.019, respectively, as seen in Table 3. The incidence of POCD using MMSE was 15.7% and statistically different from the POCD incidence of 32.9% using MoCA (p=0,018). The intraclass correlation coefficient (ICC) between the MMSE and MoCA was found to be statistically significant (p < 0.01; Table 4).

**Table 3:** Comparison of preoperative and postoperative MMSE and MoCA scores in the general and neuraxial anesthesia groups † The data are expressed as median (min-max) and the Wilcoxon test was used for comparisons. Significance levels were *p<0.05, **p<0.01, and ***p<0.001. MMSE: Mini-Mental State Examination; MoCA: Montreal Cognitive Assessment

	Preoperative score	Postoperative score	p-value†
MMSE in general anesthesia	27 (24- 30)	27 (20- 30)	0.003**
MMSE in spinal anesthesia	28 (24- 30)	27 (19- 30)	0.531
MoCA in general anesthesia	25 (18- 30)	22 (16- 29)	0.000***
MoCA in spinal anesthesia	25 (20- 30)	23 (15- 29)	0.019*

**Table 4:** Correlation of the MMSE and MoCA according to the intraclass correlation coefficient (ICC) MMSE: Mini-Mental State Examination; MoCA: Montreal Cognitive Assessment

	ICC	p-value	95% confidence interval	
MMSE & MoCA	0.789	0.000	Lower bound 0.682	Upper bound 0.864

According to the postoperative (24th hour) MoCA measurements, the incidence of POCD (MoCA < 21) was similar between the general and spinal anesthesia groups (31.9% (n = 15) and 34,7% (n = 8), respectively, with p = 0.810) and the overall incidence was 32.9% (n = 23). The gender distribution was similar between POCD and normal patients (the F/M (n%) ratios were 6/17 (21.6%/73.9%) and 7/39 (15.2%/84.8%), respectively, with p = 0.334). The mean age, duration of operation, and postoperative laboratory parameters were also similar between the POCD group (24th hour MoCA < 21) and normal group (24th hour MoCA ≥ 21) (Table 5).

**Table 5:** Comparison of patients with normal cognitive function and POCD according to age and laboratory parameters † t-test: Mann-Whitney U-test POCD: postoperative cognitive dysfunction

	Normal (Postoperative 24^th^ h MoCA ≥ 21)		POCD (Postoperative 24^th^ h MoCA < 21)		
	Mean ± SD	Median (min- max)	Mean ± SD	Median (min- max)	p value^†^
Age (years)	69.02 ± 4.18	68 (65- 82)	70.83 ± 5.05	70 (65- 85)	0.120
Duration of operation (min)	61.28 ± 21.15	60 (30- 120)	58.26 ± 21.98	55 (30- 90)	0.613
Blood glucose(mg/dL)	93.51 ± 18.09	89 (65- 148)	102.74 ± 25.39	94 (74- 167)	0.176
Urea (mg/dL)	39.52 ± 13.66	36.5(20- 77)	41.14 ± 16.04	41 (5- 93)	0.479
Creatinine (mg/dL)	1.12 ± 0.47	1.03 (0.63- 3.12)	1.04 ± 0.21	1.04 (0.69- 1.49)	0.826
Sodium (mEq/L)	138.36 ± 2.44	139 (130- 143)	132.33 ± 29.49	139 (4- 142)	0.681
WBC (µl/ml)	8.04 ± 2.31	7.8 (4.5- 14.80)	7.57 ± 2.26	7.4 (4.70- 13.70)	0.361
Hgb (g/dL)	13.64 ± 1.78	14.1 (9.5- 16.5)	13.57 ± 1.41	13.85 (9.7- 15.9)	0.661

## Discussion

The present prospective observational study of 70 patients aged ≥ 65 years undergoing elective inguinal hernia repair demonstrated a significant burden of early POCD. In a sample of 30 patients who underwent cystoscopy or hysteroscopy, Rohan et al. reported that POCD was present in 47% of patients who received propofol and 47% of patients who received sevoflurane [11]. In our observational study involving 70 patients, the incidence of early POCD was similar between the general and spinal anesthesia groups (31.9% and 34.7%, respectively, with p = 0.810), with an overall incidence of 32.9%.

Current evidence in the literature shows that there is no long-term cognitive impairment attributable to surgery and anesthesia [18,19]. However, considering that, in the early postoperative period, POCD may adversely affect the quality of life and patient outcomes, it can be assumed that cognitive screening via neuropsychiatric tests is important both for elderly patients’ outcomes and hospital costs [9].

Although many neuropsychiatric tests with different advantages have been previously used for cognitive screening, it is unclear which of them is more effective in screening for POCD. In agreement with the current literature [15-17], our study showed that the MoCA test could detect cognitive decline in both the general and spinal anesthesia groups, while the MMSE test did so only in the general anesthesia group.

When we examined the demographic characteristics of the patients with and without POCD, the anesthesia method used, duration of operation, and laboratory results, we could not find any differences between the groups. Similar results have been obtained in the literature, where no variation was observed for different intraoperative anesthesia techniques in patients with POCD [20].

Limitations

The influence of characteristics such as anxiety and sociocultural levels, which may have affected the cognitive functions of the patients, was not investigated, as we focused on medical features such as laboratory results, comorbidities, and the anesthesia method used. However, preoperative anxiety and low educational attainment have been reported to be associated with an increased risk of decline in executive function [21]. Second, the pain levels of the patients were not determined. Postoperative pain is a modifiable factor that has been shown to be associated with delirium and cognitive decline [20]. Finally, one of the important limitations of our study is the sample size disparity between the groups and the overall small sample size, since it is not a prospective randomized study.

## Conclusions

Early POCD is a frequent and important problem after elective minor surgeries in elderly patients, even in the case of spinal anesthesia. Although the MMSE is a widely used neuropsychiatric screening tool, it may not be able to detect a significant number of cases. The MoCA is an alternative tool that can be more sensitive than the MMSE in identifying cognitive decline in elderly patients undergoing minor surgeries under both general and spinal anesthesia.
